# Pessoas idosas dependentes e sua saúde mental: estudo multicêntrico brasileiro

**DOI:** 10.1590/0102-311XPT142424

**Published:** 2025-05-23

**Authors:** Girliani Silva de Sousa, Denise Machado Duran Gutierrez, Raimunda Magalhães da Silva, Amanda Márcia dos Santos Reinaldo, Meiry Fernanda Pinto Okuno, Maria Cecília de Souza Minayo

**Affiliations:** 1 Universidade Federal de São Paulo, São Paulo, Brasil.; 2 Faculdade de Educação, Universidade Federal do Amazonas, Manaus, Brasil.; 3 Universidade de Fortaleza, Fortaleza, Brasil.; 4 Universidade Federal de Minas Gerais, Belo Horizonte, Brasil.; 5 Centro Latino-Americano de Estudos de Violência e Saúde Jorge Careli, Fundação Oswaldo Cruz, Rio de Janeiro, Brasil.

**Keywords:** Idoso Fragilizado, Saúde Mental, Resiliência, Depressão, Frail Elderly, Mental Health, Resilience, Depression, Anciano Frágil, Salud Mental, Resiliencia Psicológica, Depresión

## Abstract

A dependência funcional de idosos e suas repercussões na saúde mental são desafios relevantes para as agendas de políticas públicas direcionadas para esse grupo no Brasil. Este estudo analisou as experiências relacionadas à saúde mental de idosos com dependência em cinco regiões brasileiras. Constitui investigação qualitativa, arrimada em referencial teórico-metodológico da hermenêutica-dialética, incluindo entrevistas semiestruturadas com 47 idosos em Belo Horizonte (Minas Gerais), Porto Alegre (Rio Grande do Sul), Araranguá (Santa Catarina), Manaus (Amazonas), Brasília (Distrito Federal), Fortaleza (Ceará) e Teresina (Piauí), no período de agosto a dezembro de 2019. O exame dos indicadores seguiu os passos da análise de conteúdo, obtendo as temáticas: “Quando se perde a alegria de viver” e “Recursos para o enfrentamento da vida e demanda por significado”. Idosos expressaram sintomas de depressão e ansiedade, pensamentos de morte, sensação de ser um fardo e solidão. A violência potencializa os sintomas depressivos. A resiliência, o bem-estar espiritual, a socialização, os relacionamentos interpessoais com qualidade e a religiosidade são partes dos relatos dos idosos que vivenciam bem-estar psicológico. A experiência da saúde mental de idosos dependentes está relacionada à fragilidade física e emocional, mas eles alcançam fortalecer as vertentes espirituais e religiosas. São necessárias ações do Estado para promover o cuidado qualificado e integral, a criação de espaços de atividades sociais e assistência, bem como a participação de cuidadores formais e programas de apoio em domicílio.

## Abstract (en)

The functional dependency of older adults and its repercussions on mental health represent significant challenges for public policy agendas targeting this group in Brazil. This study analyzed the mental health experiences of dependent older adults across Brazil’s five regions. It is a qualitative investigation grounded in the theoretical-methodological framework of dialectical hermeneutics, and it includes semi-structured interviews with 47 older adults conducted in Belo Horizonte (Minas Gerais), Porto Alegre (Rio Grande do Sul), Araranguá (Santa Catarina), Manaus (Amazonas), Brasília (Federal District), Fortaleza (Ceará), and Teresina (Piauí) from August to December 2019. The data were analyzed using Content Analysis, which yielded the themes “When the joy of living is lost” and “Resources for coping with life and the quest for meaning”. Participants reported depressive and anxious symptoms, thoughts of death, feelings of being a burden, and loneliness. Moreover, experiences of violence exacerbated depressive symptoms. Resilience, spiritual well-being, socialization, quality interpersonal relationships, and religion were found in the reports of older adults who experience psychological well-being. Mental health of dependent older adults is linked to physical and emotional frailty; however, they are able to reinforce their spiritual and religious resources. State actions are necessary to promote qualified and comprehensive care, create spaces for social activities and support, as well as involve formal caregivers and home support programs.

## Resumen

La dependencia funcional de las personas mayores y sus repercusiones en la salud mental son desafíos relevantes para las agendas de políticas públicas dirigidas a este grupo en Brasil. Este estudio analizó las experiencias con la salud mental de las personas mayores con dependencia en las cinco regiones brasileñas. Se trata de una investigación cualitativa, con base en un marco teórico-metodológico de la hermenéutica-dialéctica, que incluye entrevistas semiestructuradas con 47 personas mayores en Belo Horizonte (Minas Gerais), Porto Alegre (Rio Grande do Sul), Araranguá (Santa Catarina), Manaus (Amazonas), Brasília (Distrito Federal), Fortaleza (Ceará) y Teresina (Piauí), en el período de agosto a diciembre de 2019. El análisis de los indicadores siguió los pasos del análisis de contenido, del cual obtuvo los temas: “Cuando se pierde la alegría de vivir” y “Recursos para afrontar la vida y la demanda de sentido”. Las personas mayores experimentaron síntomas depresivos y ansiosos, pensamientos de muerte, sensación de ser una carga y soledad. La violencia potencia los síntomas depresivos. La resiliencia, el bienestar espiritual, la socialización, las relaciones interpersonales con calidad y la religiosidad fueron relatados por las personas mayores que han experimentado bienestar psicológico. La experiencia de salud mental de las personas mayores dependientes está relacionada con la fragilidad física y emocional, pero logran fortalecer los aspectos espirituales y religiosos. Se necesitan acciones del Estado para promover la atención calificada e integral, la creación de espacios para actividades sociales y de ayuda, así como la participación de cuidadores formales y programas de apoyo a domicilio.

## Introdução

A garantia da dignidade de vida entre os idosos com incapacidades funcionais e doenças crônicas - passíveis de conduzir à dependência - constitui uma preocupação relevante para a saúde pública no Brasil que, mormente em decorrência do incremento global da população acima de 80 anos, lida com elevadas taxas de sofrimento psíquico: a depressão, mais especificamente ^1^.

Essa realidade impõe desafios econômicos, que precisam ser compreendidos e gerenciados adequadamente para garantir a sustentabilidade e o desenvolvimento nacional. Em comparação aos brasileiros, denota-se que os idosos de nações da Europa Ocidental, Singapura, Japão, Israel e Coreia do Sul são mais saudáveis e possuem melhores indicadores de saúde ^2^.

O envelhecimento populacional constitui, sem dúvida, um fardo para a sociedade quando o Estado não logra suprir a necessidade de apoio às pessoas idosas com dependências ^2^. O choque econômico e o aumento das demandas por serviços de saúde são suscetíveis a serem maiores, ao considerar também a depressão, a ansiedade, os problemas de sono e outros transtornos mentais que acometem os dependentes sob exame. Neste estudo, o termo dependência compreende a necessidade de apoio parcial ou integral para executar as atividades básicas, instrumentais ou avançadas de vida diária ^1^.

Uma investigação encontrou associação entre a solidão e o isolamento social com maiores níveis de fragilidade entre os idosos ^3^. Esses, nos Estados Unidos, por exemplo, com três ou mais limitações físicas, relataram mais sintomas depressivos em relação àqueles com nenhuma a duas limitações. Os idosos na faixa de 75 anos ou mais revelaram terem maiores índices de depressão ^4^.

Estudos brasileiros demonstraram associações indiretas entre o maior número de morbidades, mais dependência e, consequente, menor participação em atividades básicas, instrumentais e avançadas de vida, mediadas pela fragilidade e sintomatologia depressiva ^5^ ^,^ ^6^.

O otimismo é um recurso protetor que reduz os sintomas depressivos estudados, de sorte que se encontra condicionado ao nível de estresse vivenciado pela pessoa idosa ^4^. Para estas, no entanto, as sérias limitações físicas são um fato impeditivo da capacidade de viver independentemente e de realizar as tarefas diárias necessárias ^4^.

Este estudo, pois, questiona: Como está a saúde mental das pessoas idosas brasileiras com dependência de cuidados? Ainda são limitadas - bem incipientes - no Estado Brasileiro. Pesquisas de cunho qualitativo que concedem voz aos idosos, descrevem suas experiências e as influências negativas da dependência na sua saúde mental.

Esse estudo objetiva analisar as experiências sobre a saúde mental das pessoas idosas com dependências nas mencionadas cinco regiões brasileiras e tem o intento de conceder visibilidade para a saúde mental desse contingente, configurando relevante estratégia para lutar pela qualidade de vida.

Os resultados serão suscetíveis ao apontamento de caminhos para efetivar a Política Nacional da Saúde para a Pessoa Idosa (PNSPI) ^7^, por meio de implementação de ações nos serviços de saúde de atenção primária e especializados em geriatria e gerontologia - bem como implementar a transformação das atitudes dos profissionais para o atendimento integral, acolhedor e humanizado, com suporte à compreensão das crenças e dos mecanismos comportamentais da conjunção humana necessitada de apoio.

## Método

### Tipo de estudo

Estudo qualitativo multicêntrico, com referencial teórico da hermenêutica-dialética, visando investigar os sentidos que os sujeitos atribuem aos fenômenos e ao conjunto de linguagem, símbolos e cultura em que se inserem - o que inclui as próprias contradições ^8^ ^,^ ^9^. A pesquisa recorreu ao instrumento *Consolidated Criteria for Reporting Qualitative Research* (COREQ; Critérios Consolidados para Relatórios de Pesquisa Qualitativa) ^10^ para conferência das etapas do método.

### Local de estudo

O ensaio multicêntrico relatado ^11^ realizou-se nas regiões Sudeste (Belo Horizonte/Minas Gerais), Sul (Porto Alegre/Rio Grande do Sul e Araranguá/Santa Catarina), Nordeste (Fortaleza/Ceará e Teresina/Piauí), Norte (Manaus/Amazonas) e Centro-oeste (Brasília/Distrito Federal). Essas localidades situam-se nas cinco regiões que constituem o Brasil e possuem características sociais convergentes em serviços de saúde - semelhantes para o atendimento ao idoso com dependências.

### Critérios de inclusão

Os participantes incluídos caracterizam-se por serem brasileiros, com idade de 60 anos ou mais, com algum grau de dependência funcional devidamente comprovado nos prontuários - de, no mínimo, há seis meses -, e ter capacidade verbal e orientação para responder perguntas da entrevista. Excluíram-se os que residiam em instituições de longa permanência para idosos (ILPI), ou que moravam sozinhos e que estivessem em investigação para o diagnóstico de doenças de Alzheimer ou outras demências.

Quarenta e sete pessoas idosas foram recrutadas como partícipes do experimento, tendo como suporte a estratégia de amostragem por conveniência. Nesse caso, os profissionais das unidades básicas de saúde (UBS) e dos ambulatórios especializados em geriatria as elegeram para compor o agrupamento com base nos critérios de inclusão e exclusão da pesquisa. Os serviços de saúde escolhidos nas cidades brasileiras possuem aproximações com os pesquisadores envolvidos no estudo por serem campos de práticas de ensino e extensão.

### Coleta de dados

As entrevistas foram realizadas no período de agosto a dezembro de 2019. Entre os entrevistados não ocorreram desistências. Inicialmente, os profissionais de saúde entregaram uma carta-convite às pessoas idosas, e, posteriormente, foi agendado o melhor dia para as entrevistas semiestruturadas, que ocorreram presencialmente em suas residências. No início, os entrevistadores mantiveram uma conversa informal, apresentaram a dupla que conduziria a entrevista, explicaram os objetivos da pesquisa e foram observados os critérios de inclusão. O tempo de entrevista variou entre 30 e 60 minutos. O Termo de Consentimento Livre e Esclarecido foi lido integralmente e assinado em duas vias. Utilizou-se um roteiro semiestruturado de entrevista, elaborado pelos pesquisadores em uma oficina participativa presencial ^11^. As entrevistas foram conduzidas por uma dupla de pesquisadores-seniores idosos e jovens acadêmicos vinculados às instituições de Ensino Superior (IES) com experiência em pesquisas qualitativas. Todos os entrevistadores realizaram treinamento em dupla para se capacitarem sobre o uso do roteiro de entrevista semiestruturada, com vistas a que esses instrumentos ocorressem de modo respeitoso, acolhedor e sem julgamento. Essa investigação trouxe o recorte com a pergunta: “Como você se sente diante da sua dependência de cuidados em relação aos seus familiares?” e, com base nesta indagação, foram sendo elaboradas outras questões que possibilitaram ao entrevistado aprofundar a temática. Os áudios foram gravados e transcritos integralmente, com a permissão das pessoas entrevistadas.

### Análise dos dados

Os indicadores foram categorizados de acordo com a análise temática ^12^: transcrição dos depoimentos dos participantes na íntegra; leitura e releitura cuidadosa das entrevistas para compreensão dos sentidos atribuídos à saúde mental das pessoas idosas dependentes de cuidados; identificação de trechos que englobavam sentidos comuns das vivências e experiências dos participantes; e organização dos relatos em categorias temáticas que manifestassem o dito e o não dito das emoções. Recorreu-se ao emprego da triangulação de dados com a hermenêutica-dialética ^8^ ^,^ ^9^, da literatura científica e das interpretações dos pesquisadores para realizar uma análise compreensível e cuja discussão dos resultados ressaltaram: “Quando se perde a alegria de viver” e “Recursos para o enfrentamento da vida e demanda por significado”.

### Aspectos éticos

O estudo seguiu as diretrizes da *Resolução nº 466/2012* e da *Resolução nº 510/2016*, do Conselho Nacional de Saúde (CNS) e obteve aprovação do Comitê de Ética em Pesquisa da Escola Nacional de Saúde Pública Sergio Arouca, Fundação Oswaldo Cruz (CAAE: 44615315.0.0000.5240, parecer nº 1.326.631). As narrativas estão expressas por meio das iniciais do nome do entrevistado, seguidas pela identificação de idade, sexo, orientação sexual, doença, nível de dependência e cidade.

## Resultados

Em sua maioria, os idosos participantes estavam na faixa dos 80 anos ou mais - um grupo composto majoritariamente por viúvas, acometidas por acidente vascular cerebral e com necessidade de cuidados há mais de três anos. As condições sociodemográficas dos participantes estão descritas no Quadro 1.

**Quadro 1:** Caracterização sociodemográfica de idosos dependentes de cuidados. Brasil, 2024.

IDADE (ANOS)	SEXO	ESTADO CIVIL	CIDADE	DOENÇA	TEMPO DE DEPENDÊNCIA
87	Feminino	Viúva	Araranguá	Câncer de mama	1 ano
84	Feminino	Viúva	Araranguá	Acidente vascular cerebral e queda	3 anos
80	Feminino	Viúva	Araranguá	Parkinson	9 anos
75	Feminino	Viúva	Araranguá	Acidente vascular cerebral e queda	7 anos
88	Feminino	Viúva	Araranguá	Acidente vascular cerebral	10 anos
88	Feminino	Viúva	Araranguá	Acidente vascular cerebral	1 ano
90	Masculino	Viúvo	Araranguá	Artrite	8 anos
99	Masculino	Viúvo	Araranguá	Artrite	3 anos
72	Masculino	Casado	Araranguá	Acidente vascular cerebral e câncer de próstata	6 meses
89	Masculino	Viúvo	Araranguá	Acidente vascular cerebral	5 anos
80	Masculino	Viúvo	Araranguá	Glaucoma seguido de cegueira	10 anos
84	Masculino	Casado	Araranguá	Acidente vascular cerebral	5 anos
79	Feminino	Divorciada	Belo Horizonte	Acidente vascular cerebral	6 anos
75	Feminino	Viúva	Belo Horizonte	Dor crônica, artrose e diarreia crônica	2 anos
81	Feminino	Viúva	Belo Horizonte	Acidente vascular cerebral	3 anos
66	Feminino	Viúva	Belo Horizonte	Fratura na coluna	4 anos
83	Feminino	Casada	Belo Horizonte	Câncer de pulmão e artrite	2 anos
69	Masculino	Divorciado	Belo Horizonte	Doença de Parkinson	3 anos
65	Masculino	Casado	Brasília	Acidente de carro e perda de movimentos	30 anos
81	Feminino	Casada	Brasília	Acidente vascular cerebral	21 anos
69	Feminino	Viúva	Brasília	Acidente vascular cerebral	3 anos
67	Masculino	Casado	Brasília	Fratura no joelho	10 anos
67	Masculino	Casado	Brasília	Doença de Parkinson	15 anos
72	Masculino	Casado	Brasília	Acidente vascular cerebral	9 anos
70	Feminino	Viúva	Fortaleza	Acidente vascular cerebral	6 anos
89	Feminino	Viúva	Fortaleza	Pé diabético	5 anos
77	Feminino	Viúva	Fortaleza	Artrose e diabetes	8 anos
85	Masculino	Casado	Fortaleza	Fratura no quadril	1 ano
76	Feminino	Casada	Fortaleza	Demência	3 anos
93	Feminino	Viúva	Manaus	Fratura no quadril	5 anos
83	Feminino	Viúva	Manaus	Glaucoma seguido de cegueira	7 anos
79	Feminino	Viúva	Manaus	Hanseníase e problemas de locomoção	5 anos
96	Feminino	Viúva	Manaus	Glaucoma seguido de cegueira	3 anos
70	Masculino	Casado	Porto Alegre	Atrofia e diabetes	1 ano
74	Feminino	Casada	Porto Alegre	Câncer na medula	3 anos
67	Feminino	Viúva	Porto Alegre	Fratura no joelho	2 anos
80	Masculino	Casado	Porto Alegre	Neuropatia e diabetes	10 anos
75	Masculino	Casado	Porto Alegre	Insuficiência renal e artrose	3 anos
82	Feminino	Viúva	Porto Alegre	Câncer no cérebro	6 meses
89	Feminino	Viúva	Porto Alegre	Problema vascular na perna	3 anos
75	Feminino	Viúva	Porto Alegre	Câncer no estômago	3 anos
72	Feminino	Viúva	Porto Alegre	Artrite e diabetes	1 ano
64	Feminino	Divorciada	Porto Alegre	Insuficiência renal	6 meses
60	Masculino	Casado	Teresina	Acidente vascular cerebral	6 meses
83	Feminino	Viúva	Teresina	Artrite e câncer no estômago	3 anos
87	Feminino	Viúva	Teresina	Artrose	3 anos
86	Feminino	Viúva	Teresina	Insuficiência cardíaca e vasculite	6 anos

### Quando se perde a alegria de viver

Nessa temática, foram englobadas diversas experiências que expressaram o esgotamento da vitalidade física e psíquica, a angústia de se sentir inútil e os pensamentos de morte, isolamento e solidão dos entrevistados.

A tristeza assume múltiplos sentidos para os idosos, ancorada na falta de autonomia para executar as atividades rotineiras e na vergonha das alterações no corpo que levam à limitação funcional e à solidão.

“*...do nada eu começo a chorar porque é ruim ficar parada, eu gosto de ver gente... No começo foi muito ruim, eu sentia vergonha, comecei a não aceitar a bengala, a última vez que esses meninos* [profissionais de saúde da atenção primária] *estiveram aqui, eles falaram que eu tenho que andar de cadeira de rodas, eu falei nem de cadeira e nem de roda*” (C.S.S., 75 anos, mulher, cis heterossexual, branca, viúva, dependente parcial, dor crônica, artrose e diarreia crônica há dois anos, Belo Horizonte).

A sensação de inutilidade aparece com relevância e a percepção de perdas entre um tempo anterior de controle sobre a vida e autossuficiência, e a velhice que exprime a dificuldade de pedir ajuda para seus familiares, são sentidos entrelaçados - demarcados por tristeza, nervosismo e ansiedade. A irritabilidade foi verbalizada pelos idosos, tanto homens quanto mulheres, em todas as regiões.

“*...eu nunca dependi de ninguém, com 15 anos meu pai morreu e eu virei chefe da família, cuidei dos meus irmãos e eu comandei o espetáculo e agora com essa doença* (...) *tem horas que do nada vem o choro, tu fica deprimido, pensativo e quando vê os olhos já estão molhados, eu não sei se isso é fraquejar, eu nunca quis chorar, sempre tento ser um cara forte, mas eu tô vendo que eu não sou essa fortaleza, sinto uma angústia por dentro muito grande, eu não quero sofrer, eu já tive ataques de pânico e é uma coisa horrível, pior do que a dor*” (F.S.D., 70 anos, casado, homem, pardo, heterossexual, atrofia e diabetes há um ano, Porto Alegre).

O adoecimento com dor se torna um martírio, constituindo um sofrimento capaz de ser pior do que morrer. Os pensamentos negativos e a frustração culminam na sensação de ser um fardo - e com isso, o desejo da morte.

“*...penso em suicídio, não aceito minha situação, eu fico nervoso por não poder ajudar em casa, por exemplo, arrumar uma torneira vazando, me sinto incapaz, não tem o que fazer, as pernas não aguentam andar, me sinto inseguro em sair de casa, é muito ruim perder o controle dos meus movimentos*” (R.R., 75 anos, homem, cis heterossexual, casado, pardo, com diagnóstico de doença de Parkinson há cinco anos, Brasília).

A baixa autoestima, juntamente com a tristeza, estão no relato dos circunstantes examinados, que tiveram outros adoecimentos no decurso da vida. A violência psicológica e o abandono potencializaram os sintomas depressivos.

“*...eu já fui muito discriminada no passado quando tive hanseníase, as pessoas mudavam de calçada. Hoje, me sinto triste, os outros me humilhando*” (C.M., 76 anos, mulher, cis heterossexual, branca, casada, fratura na coluna, dependência total há cinco anos, Manaus).

A solidão aparece como um apagamento de si dentro da sociedade. Os dias são longos e solitários em um encontro com a própria identidade, soterrando esperanças e fazendo com que os idosos se sintam frágeis. Possivelmente, a maior frustração não seja a dependência, mas a sensação de não ter com quem compartilhar ideias e pensamentos.

“*Só fico aqui na rede deitado, porque depois da queda, ficou difícil de andar, eu fico isolado aqui porque não tenho ninguém para conversar. O que eu sinto vontade é de ir para a rua, passear, mas não tem quem me leve*” (A.L.S., 85 anos, homem cis heterossexual, pardo, casado, dependente físico há dois anos após queda, Fortaleza).

Os sintomas depressivos expressos pela dor psicológica estão numa quantidade significativa de partícipes, os quais manifestam dependência física acompanhada de dor crônica.

“*...é uma tristeza que dói lá dentro, já passou tanto tempo que a gente até se acostuma, eu acho a vida sem graça, os outros ficam empurrando a gente em um carrinho, não é preconceito, é que eu gosto de tá me movimentando, mas agora eu sinto dor, ela vem de repente, como é que uma pessoa que não dá conta de caminhar e sente dor vai ter alegria?*” (J.S.R., mulher, 75 anos, viúva, cis heterossexual, branca, câncer no pulmão e dificuldade de locomoção, dependente há oito anos, Belo Horizonte).

### Recursos para o enfrentamento da vida e demanda por significado

Nessa temática, exploram-se os modos de os idosos desenvolverem estratégias para o enfrentamento da vida, como experiências espirituais, resiliência e relacionamentos.

Homens e mulheres idosas demandaram por outras maneiras de se sentirem úteis, com flexibilidade cognitiva para criar uma rotina diferente. A resiliência e a resignação foram estratégias para lidar com a dependência e o adoecimento. O apoio emocional e instrumental, por via da ação comunicativa e mediante a preservação da autonomia fez com que os sujeitos tivessem o senso de pertencimento e utilidade para a família.

“*...eu continuo sendo a matriarca, no final das contas, quem é que dá as ordens, quem organiza a casa e quem diz como as coisas devem ser sou eu, meus filhos me procuram ‘mãe, isso é assim?’ então eu acho que ainda sirvo para orientar alguma coisa.* (...) *a gente não pode ter tudo o que quer, tem que se conformar com o que Deus conseguiu para a gente e dar paciência para conseguir andar naquele ritmo, eu não tenho mais aquela disposição para andar, passear, fazer exercício, viajar, eu não tenho coragem de andar sozinha e de viajar, tenho medo de ter uma queda, um escorrego, a gente anda mais devagar. O que me deixa feliz é eu ter minha bodega* [comércio]*, todo mundo me conhece, um vem, conversa*” (Z.L.D., 77 anos, mulher, cis heterossexual, parda, viúva, artrose e diabetes há oito anos, dependente parcial, Fortaleza).

No ensaio sob comentários, foram encontrados seres resilientes, resignados e alegres, apesar das limitações funcionais. Homens e mulheres idosos(as), cegos(as) e acamados(as), discorreram a respeito da sua subjetividade e existência corporal com muito bom humor, de sorte que, em suas conversas, o corpo envelhecido não se transformou em um elemento de incômodo, mas compôs uma nova experiência de si.

“*...eu me sinto muito bem, fiquei cego faz muito tempo e agora estou acamado, mas procurei ver o que eu consigo fazer, quando o tempo está bom, vou pegar sol e converso com os vizinhos na calçada, quando chove, sempre aparece alguém para conversar aqui*” (O.M.T., 80 anos, homem, cis heterossexual, casado, branco, neuropatia e diabetes há 10 anos, Porto Alegre).

A busca de realização de atividades do cotidiano e a religiosidade apareceram como circunstâncias que potencializaram o bem-estar e a resiliência dos idosos/dependentes.

“*Venho me sentindo bem, sou uma velhinha conservada, eu pego alguma coisa para costurar, para me distrair, eu não posso viver parada, o juízo da gente é muito importante. Eu rezo tanto para Nossa Senhora conservar a minha cabeça boa*” (M.G.L.S., 87 anos, mulher, branca, cis heterossexual, viúva, artrose nas pernas e na coluna, dependência física, Teresina).

“*No começo me dava uma tristeza, mas tem que se acostumar porque a gente é assim tem tempo que pode trabalhar tem tempo que não pode e eu digo: Senhor se eu fiquei assim me dá força e coragem para eu viver minha vida até o dia que ele quiser, o amor de Deus é o que fortalece dentro de nós*” (R.M.S., 83 anos, mulher, parda, cis heterossexual, viúva, cega há três anos, dependente total, Manaus).

A preservação da autonomia mostra-se como uma certeza da potencialidade: aceitar com resiliência o que parece ser o destino é um ato entendido como indicativo de coragem. O prazer de ter um projeto individual - envolvendo atividades como cuidar da casa, passear, viajar - foi substituído pela satisfação de contribuir para um projeto coletivo da família.

“*Ela cuida de mim com carinho, deixa eu fazer o que eu consigo sozinho, me leva no banco para eu pegar meu dinheirinho* (...) *As pernas são bambas, não são mais pernas de pessoa jovem. Me sinto muito bem, minha família me ajuda. Quando tem sol, eu vou para a calçada, eu tenho um amigo aqui, eles vêm aqui, a gente bate papo*” (C.P., 99 anos, homem, branco, viúvo, cis heterossexual, artrose nos joelhos, anda de cadeira de rodas há dez anos, Araranguá).

## Discussão

Os grupamentos ora examinados constituem agentes sociais que, desde a infância, possuíam responsabilidades e viam os mais velhos com vitalidade, autonomia e funcionalidade. O adoecimento e a dependência rompem a perspectiva simbólica de que a velhice se constitui de saúde e disposição, comparável à juventude ^13^.

As experiências vividas por esses idosos correspondem a verdadeiras estruturas de identidade ^8^ ^,^ ^9^. Nesse sentido, veem as limitações como um luto, não apenas do corpo, mas de sua personalidade ^13^ ^,^ ^14^ ^,^ ^15^. É a pessoa idosa que se destituiu da existência laboral e de alguns prazeres, como tocar violão ou sanfona e fazer viagens com a família, para uma vida de renúncias e de anulação de possibilidades: do bem-estar corporal para o estado permanente de marcas oriundas de lesões e/ou doenças.

As perdas afloram, não apenas do ponto de vista físico, mas também da disposição mental e emocional. A tristeza foi manifestada através do choro, do mau humor, da resistência à mudança - em razão do desânimo -, conduzida por falta de energia e de interesse, mediante alterações no padrão de sono, perda de apetite e sentimento de desesperança ^13^ ^,^ ^16^. Resultados semelhantes foram encontrados em estudo relacionado aos chineses, cujas limitações funcionais estão negativamente associadas à infelicidade, e quanto esse fato prejudica a qualidade do relacionamento com os filhos adultos - por aumentar a carga de cuidados ^17^ e haver o consequente prejuízo na privacidade e autonomia dos idosos.

A tristeza assume dimensões multifatoriais em consequência do acúmulo de anos com doenças crônicas, dor arraigada, perda da autonomia e, em alguns casos, por maus-tratos recebidos e dependência física. O fato de não poder andar aniquila as possibilidades de ter esperança em um futuro melhor e isola o idoso do mundo - seja pela falta de convites, ou por uma solidão decorrente da dificuldade de aceitar a nova condição e de compartilhar isso com familiares, amigos e profissionais de saúde ^13^ ^,^ ^14^ ^,^ ^15^ ^,^ ^18^ ^,^ ^19^ ^,^ ^20^. Em outras narrações, a solidão aparece como um sinal de abandono, tanto pelas relações não terem qualidade quanto pela condição da família, na qual a organização do cuidado recai sobre um membro familiar, resultando na “infantilização” do idoso - que passa a ser considerado como destituído de intentos, vontades e sente que seus direitos foram violados ^16^ ^,^ ^19^.

A depressão, por exemplo, está associada à solidão entre pessoas idosas holandesas ^14^. O estado depressivo tem efeito negativo nos pensamentos e sentimentos e, portanto, é provável que a solidão coexista mais facilmente ^14^. Isso enseja explicar o fato de que, apesar de algumas pessoas idosas com sintomas depressivos estarem medicadas, expressarem melhoras no sono e serem acompanhadas por cuidadores familiares, os sintomas depressivos não denotam melhoras.

A solidão, muitas vezes, aumenta a perda do senso de pertencimento e impõe maior ideia de frustração ^16^ ^,^ ^20^. O isolamento é descortinado como responsável pelo aumento da impressão de aprisionamento e precursor de ideações suicidas ^20^. Atualmente, há um padrão piramidal de vulnerabilidade à solidão que ajuda a orientar políticas e intervenções ^21^. Esse modelo descreve três níveis de vulnerabilidade: (i) primária, em que a pessoa está em risco de isolamento; (ii) secundária, quando começa a se desconectar e se isolar; e (iii) terciária, onde ela se torna altamente isolada ^21^.

Ao considerar as diretrizes da PNSPI ^7^, o cuidado ofertado à saúde do idoso não deve se iniciar apenas na idade cronológica de 60 anos ou mais - mas sim, valorizar a formulação dos projetos de vida entre jovens adultos na atenção primária para a continuidade do cuidado no envelhecimento e socialização. Tal conjunção de atos exige a educação permanente aos profissionais por intermédio de uma ação comunicativa ^8^ ^,^ ^9^ que escute as demandas psicossociais do grupo etário neste exame.

Também, a doença física crônica esteve associada à depressão em pessoas idosas inglesas ^22^. É possível que, ao se sentirem deprimidas, experimentem mudanças na cognição social e, por isso, passam a se retrair socialmente ^18^, o que é capaz de justificar o “ficar quieto” das pessoas idosas. As que mais precisam de interação quando se sentem solitárias possuem menos oportunidades de conexão ou de regulação emocional interpessoal eficaz, por ser difícil compartilhar a própria experiência de solidão ^18^.

Pensamentos de morte e ideias suicidas foram identificados nos idosos deprimidos. A investigação identificou, entre os idosos, a sensação de ser um fardo, principalmente, para os cuidadores familiares mais jovens, pois são vistos como sobrecarregados ^15^ ^,^ ^23^. O fracasso é conducente a sentimentos de ineficácia ou incompetência, acarretando mais sofrimento psicológico ^24^.

A ansiedade e o estado de saúde estão associados intensamente ao medo de ser um fardo, comparativamente à depressão ^25^ ^,^ ^26^. Uma possível explicação, está nos sintomas gerais de ansiedade - como apreensão e nervosismo -, que são mais difusos e estão relacionados com preocupações orientadas para o futuro ^25^ ^,^ ^26^.

É relevante compreender que não deve ser considerado normal o fato de pessoas idosas com limitações funcionais demonstrarem desesperança ^16^, quietude e solidão, pois esse comportamento é habilitado a aumentar o risco autolesivo. Uma revisão sistemática encontrou a prevalência de 25,8% de ideação suicida em pessoas que sofrem com dores crônicas ^27^. Percepção de piora na saúde física, depressão e transtorno de estresse pós-traumático ^27^ estão associados à necessidade de se mover ou agir de modo diferente, em consequência da dor e, com isso, são criadas lacunas de identidade ^15^. O distanciamento dos idosos em relação aos ambientes sociais e à socialização contribui para que se vejam longe do senso de pertencimento, em que perdem o poder de afetar seu ambiente ^15^ ^,^ ^20^. É possível que a dificuldade de aceitar as limitações funcionais e pedir ajuda esteja associada às razões que incluem a incapacidade de os componentes familiares e amigos compreenderem a extensão dos desafios impostos à saúde, como resultante da incapacidade física. Foi comum a este estudo, identificar a falta de flexibilidade dos ambientes em serem inclusivos e adequados às atividades que as pessoas idosas costumavam fazer ou apreciar.

Evidencia-se, então, a necessidade de promover espaços inclusivos para que a pessoa idosa, copartícipe da comunidade, tenha voz reconhecida e necessidades amplificadas para mudar o processo de trabalho dos serviços de saúde, ainda centrados nas ações curativas. Impende transformar a própria realidade de exclusão em ações de promoção da saúde intersetoriais ^7^.

Chama-se a atenção para a questão da desigualdade social no Brasil. A vulnerabilidade social pode ser representada, entre tantas características, pelas casas construídas em morros com escadas íngremes - que dificultam o acesso às ruas - e passagens inviáveis às cadeiras de rodas, por exemplo. Ambos representam, simbolicamente, as limitações que o mundo ocidental impõe às pessoas idosas e/ou com limitações físicas: tais conjunturas perpassam a vida delas e as restringem às suas casas, cadeiras e/ou camas, corroborando para o sentimento de impotência, perda da autonomia e de vergonha. Em expressas circunstâncias, sair de casa se torna muito difícil, pois requer ajuda de outrem, que tenham compleição física para assegurar que o dependente transite por espaços não projetados a ele - como em ruas inadequadas para locomoção de cadeira de rodas/andadores, escadas, transporte público limitado etc. -, fazendo com que dependam da solidariedade de indivíduos para um deslocamento em veículos particulares - melhor via para a mobilidade do idoso e seu cuidador.

No estudo foram encontradas apenas idosas que sofriam algum tipo de violência - como negligência, abandono e maus-tratos. A falta de apoio social pela qual passam, aumentam essas violências, tanto na questão dos abusos psicológicos e físicos quanto nas negligências ^28^ ^,^ ^29^. Idosos bem longevos, com doenças crônicas e sintomas depressivos, são mais propensos à violência psicológica ^28^. O maior risco de ultrajes físicos está intimamente relacionado à atmosfera emocional entre os membros da família ^28^ ^,^ ^29^.

Nessa investigação, foram entrevistados idosos resilientes e com bem-estar psicológico que se acreditam capazes de solucionar problemas, lidar com desafios, sentindo-se habilitados a reconhecer e celebrar as conquistas, malgrado as dificuldades e limitações que possuem, decorrentes da dependência ^30^ ^,^ ^31^. Esses indicadores corroboram as hipóteses propostas em investigação estadunidense, em que a resiliência é propícia a ser uma elaboração multidimensional para a redução de sintomas e gravidade da depressão e diminuição das ideias de suicídio entre idosos ^32^.

Investigação nos Estados Unidos identificou que idosos mais resistentes são favoráveis a exibir características resilientes relacionadas a conflitos sociais e dificuldades pessoais ^31^. A resiliência teve associação positiva com a preservação da função cognitiva ^33^. Os profissionais de saúde estão propícios a implementar intervenções para promover a resiliência desse grupo etário.

O senso de pertencimento aufere outros contornos quando os componentes familiares valorizam a experiência dos mais velhos. Na oportunidade em que o cuidado não é imperativo e o idoso consegue - e lhe é permitido - compartilhar atividades, ele interpreta suas limitações funcionais de maneira mais positiva por via do aceite de sua condição de fragilidade. O esteio intergeracional ^30^ ^,^ ^31^, especialmente a dimensão do apoio emocional ^30^ ^,^ ^31^, esteve associado à redução dos sintomas depressivos entre idosos americanos ^31^. O suporte emocional entre gerações tem curso em países onde as famílias são responsáveis pelos cuidados de seus compartes familiares e, desse modo, corresponde às expectativas culturais tradicionais de respeito e obediência ^30^ ^,^ ^31^.

Os dados fazem com que se veja o envelhecimento com felicidade, não obstante as limitações funcionais, pois se constitui no cotidiano como uma transformação permanente de tornar-se outro, desde uma metamorfose que exige a perspectiva pessoal e coletiva - a fim de o idoso ter o suporte necessário para se reconhecer na qualidade de sujeito, dentro de suas possibilidades e limitações -, até o vislumbre do que ainda é possível de se realizar. Os comportamentos instrumentais de apoio por parte dos filhos, como fazer as tarefas domésticas e aceitar que os idosos ajudem a cozinhar, mostraram-se particularmente importantes para que eles não se achem meramente objetos de preocupação, cuidado e exclusão, mas, contrariamente, se considerem incluídos, valorizados e respeitados ^30^.

Por conseguinte, ter com quem conversar foi algo expresso como efetivo para o bem-estar dos idosos com dependência. A qualidade e a dedicação às relações sociais foram fundamentais para que eles desfrutassem e se sentissem em boa companhia e amados em seus corpos adoecidos. Esses resultados são corroborados por pesquisa com afro-americanas e sul-coreanas, em que a qualidade do relacionamento familiar, caracterizado por afeto e confiabilidade, confirma um dos fatores protetores fundamentais para a não ocorrência, piora e persistência da dor crônica à proporção do tempo ^31^ ^,^ ^32^ ^,^ ^33^.

Nessa perspectiva, a convivência com crianças, o fato de residirem na casa da família e a visita de grupos religiosos, bem como a mobilização de familiares na socialização das pessoas idosas, são aspectos importantes a fim de elas estabeleçam para si um novo modo de existência e cuidado. A participação social, o vínculo emocional e a solidariedade intergeracional possuem influxo protetor significativo sobre o bem-estar subjetivo, e são efeitos positivos na saúde dessas pessoas, segundo estudos da Índia ^32^. O Estado brasileiro tem caminhos promissores que podem oferecer uma implementação de políticas que motivem os longevos a participarem de várias atividades sociais formais e informais, com o adjutório dos profissionais de saúde em uma perspectiva interprofissional ^7^. De fato, isto beneficiaria a promoção do bem-estar subjetivo entre os idosos com laços fracos ou até ausentes relacionados à família, ou os que estão em vulnerabilidade social - e seria um suporte aos cuidadores familiares que, sozinhos, desempenham este trabalho. Não foram encontradas especificidades na maneira de lidar com a dependência entre as variadas regiões brasileiras, o que é explicado pelo fato de todos os entrevistados serem usuários do Sistema Único de Saúde (SUS), com similaridade de situação econômica, residência e configuração familiar.

A religiosidade também aparece como uma bússola que os direciona a uma vida definida por acolhimento de si mesmo e suas condições. Essa vivência está em sintonia com pesquisa da psicologia positiva, no âmbito da qual ter práticas religiosas e ser religioso têm uma relação positiva de satisfação com a vida, com o emprego frequente de pontos fortes e a percepção de se sentir realizado ^34^. O bem-estar espiritual concede oportunidade a uma adaptação melhor às reais limitações funcionais e fortalece o idoso para entender que a vida se rearranja para compensar perdas. Uma investigação na Alemanha descobriu que o envolvimento em práticas religiosas foi associado a pontos fortes de caráter: gratidão, bondade e perdão ^35^. Assim, na cultura brasileira, o contato com grupos religiosos e momentos de práticas da espiritualidade constituem estratégias para a manutenção do possível bem-estar do idoso.

## Considerações finais

É oportuno evidenciar que esta investigação exprime limitações. A experiência de adoecimento e as repercussões psicossociais, temporalmente, não lograram ser compreendidas e tampouco foi elucidada a influência da cultura, da religião e do gênero de cada região do Brasil. Pesquisas, doravante, são propícias a investigar as constantes transformações e a evolução no prognóstico de variadas doenças e a saúde mental de pessoas idosas que necessitam de cuidados.

As experiências desses compartes da sociedade, necessitados de atenção na questão de saúde mental, indicam os caminhos para a efetivação das diretrizes da PNSPI ^7^, e os resultados encontrados apontam para a necessidade da promoção de relações interpessoais por parte de profissionais de saúde da atenção primária e especializada, ao valorizarem as vozes dos idosos que ecoam para compreender - com solidariedade, empatia e compaixão - a existência de sintomas depressivos, a dificuldade de aceitar as limitações funcionais, o constrangimento ao pedir ajuda e a sensação de ser um fardo em um caminho para alcançar a atenção integral à saúde da pessoa idosa.

Os idosos valorizam a dimensão afetiva das relações interpessoais com amigos, vizinhos e familiares. A funcionalidade corporal ocupa pouco espaço na cultura local, quando se divisa que a socialização e a habilidade comunicativa anunciam a potência da pessoa idosa - revelando que a condição de incapacidade funcional não as define. Aquelas que se sentem bem e esperançosas, não com uma melhora e retorno ao controle do corpo, mas com esperança de viver conscientemente - e tomadas da sensação de bem-estar e felicidade - procuram se sentir úteis com tarefas que lhes são possíveis e razoáveis. O bem-estar espiritual, a socialização com vizinhos e grupos religiosos, bem como a própria religiosidade, constituem fundamentos do bem-estar físico, emocional, espiritual, e, ainda, do bem viver da população nacional longeva. Os gestores e profissionais de saúde devem promover ações intersetoriais para fomentar a participação dos idosos nos grupos operativos e nos grupamentos de convivência, considerando as limitações funcionais e a aposta na troca de experiências nesse contingente etário para a promoção do envelhecimento saudável.
